# Implication of Urinary Complement Factor H in the Progression of Immunoglobulin A Nephropathy

**DOI:** 10.1371/journal.pone.0126812

**Published:** 2015-06-02

**Authors:** Maojing Liu, Yuqing Chen, Jingjing Zhou, Ying Liu, Fengmei Wang, Sufang Shi, Yanfeng Zhao, Suxia Wang, Lijun Liu, Jicheng Lv, Hong Zhang, Minghui Zhao

**Affiliations:** 1 Renal Division, Department of Medicine, Peking University First Hospital; Beijing 100034, China; 2 Institute of Nephrology, Peking University; Beijing 100034, China; 3 Key Laboratory of Renal Disease, Ministry of Health of China; Beijing 100034, China; 4 Key Laboratory of Chronic Kidney Disease Prevention and Treatment, Ministry of Education; Beijing 100034, China; 5 Laboratory of Electron Microscopy, Peking University First Hospital, Peking University First Hospital; Beijing 100034, China; Mario Negri Institute for Pharmacological Research and Azienda Ospedaliera Ospedali Riuniti di Bergamo, ITALY

## Abstract

**Background:**

After activation, the complement system is involved in the pathogenesis of Immunoglobulin A nephropathy (IgAN). Complement factor H (CFH) is a crucial inhibitory factor of the alternative pathway of the complement system. The study investigated the effects of urinary CFH levels on IgAN progression.

**Methods:**

A total of 351patients with IgAN participated in this study. They were followed up for an average of 51.8±26.6 months. Renal outcome was defined as a composite endpoint, that included instances of end-stage renal disease (ESRD),≥ 50% decline in estimated glomerular filtration rate (eGFR) or doubling of plasma creatinine levels. Urinary CFH levels were measured by enzyme-linked immunosorbent assay and calculated as the ratio of urinary CFH over creatinine (uCFH/uCr).

**Results:**

In the whole cohort, uCFH/uCr values were associated with disease progression either as continuous [log(uCFH/uCr)] or categorical traits (dichotomous and quartile variables) after adjusting for eGFR, proteinuria, mean arterial blood pressure, histological grading and immunosuppressive therapy in the Cox proportional hazard model. Kaplan-Meier analysis showed that higher uCFH/uCr values at baseline predicted worse renal outcome during follow-up (log-rank, *P*<0.001). Receiver operating characteristic curve (ROC) analysis showed that log(uCFH/uCr) had predictive value for renal outcome (area under curve [AUC]=0.745), and the AUC increased to 0.805 after being incorporated into baseline eGFR and proteinuria. In subgroup analysis with eGFR≥60 mL/min/1.73m^2^, log(uCFH/uCr) had better predictive value (AUC= 0.724, *P*=0.002) for renal outcome compared to eGFR (AUC = 0.582, *P*=0.259) and proteinuria (AUC = 0.615, *P*=0.114).

**Conclusions:**

Urinary CFH levels are associated with renal function decline and increased urinary CFH levels are a risk factor for progression of IgA nephropathy.

## Introduction

Complement factor H (CFH) is one of the crucial inhibitory factors of the alternative pathway of the complement system, acting to accelerate inactivation of C3b [1].Studies suggest that activation of the complement alternative pathway may be involved in Immunoglobulin A nephropathy (IgAN) pathogenesis [2,3,4,5,6,7,8,9,10,11]. Our previous study and results from other studies demonstrated deposition of CFH and C3b in renal tissue of patients with IgAN, and higher levels of serum/urinary CFH were associated with disease activity [12,13,14,15]. However, it has remained unknown whether urinary CFH levels are associated with disease progression.

In this study, we measured urinary CFH levels in our IgAN cohort, analyzed the correlation between these levels and clinical/histological parameters, and evaluated the value of urinary CFH levels for predicting renal outcome.

## Materials and Methods

### Subjects

This study was approved by Clinical Research Ethics Committee of Peking University First Hospital. All individuals included in this study signed consent forms so that their information could be stored in the hospital database and used for research.

A total of 351patients with IgAN were included in this study. Patients who had participated in a previous CFH study were not included [15].Clinical parameters, including age, gender, blood pressure, proteinuria, plasma creatinine levels and other physical and biochemical characteristics were collected on the day of renal biopsy and during follow-up. The estimated glomerular filtration rate (eGFR) was calculated using the Chronic Kidney Disease Epidemiology Collaboration equation [16]. Renal pathological lesions were graded according to the Hass classification system [17]. Histological grades were divided into groups A (Hass I, II, and III) and B (Hass IV and V) [18] (Table 1).

**Table 1:** Baseline characteristics of the IgAN cohort.

	The whole group	uCFH/uCr Low	uCFH/uCr High	P value	The subgroup (eGFR≥60ml/min/1.73m^2^)	uCFH/uCr Low	uCFH/uCr High	P value	The subgroup (eGFR<60ml/min/1.73m^2^)	uCFH/uCr Low	uCFH/uCr High	P value
**N**	351	176	175		280	140	140		71	36	35	
**Gender *(Male)***	183(52.0%)	95(53.9%)	88(50%)	0.489	133(47.5%)	71(50.7%)	62(44.3%)	0.338	50(70.4%)	26(72.2%)	24(68.6%)	0.741
**Age *(year)***	33.6±11.8	32.1±10.4	35.2±12.8	0.013	32.1±11.2	31.0±9.9	33.1±12.3	0.12	39.7±11.9	40.8±9.7	39.4±13.9	0.818
**MAP *(mm Hg)***	93.6±11.4	92.6±10.8	94.6±11.9	0.101	92.0±11.1	92.1±10.9	91.9±11.3	0.885	100.0±10.4	99.4±10.6	100.7±10.4	0.579
**Proteinuria *(g/d)***	1.56 (0.03–16.40)	1.1 (0.03–14.8)	2.3 (0.2–16.4)	<0.001	1.33 (0.09–16.40)	1.1 (0.1–14.8)	1.8 (0.2–16.4)	<0.001	2.4 (0.03–13.8)	1.8 (0.03–7.83)	2.4 (0.03–13.8)	<0.001
**eGFR *(ml/min/1*.*73m2)***	85.95±29.69	94.5±26.2	76.9±30.3	<0.001	96.93±21.72	100.4±21.7	93.4±21.2	0.007	42.6±12.1	45.4±10.7	39.7±12.7	0.045
**CKDstage *1/2/3/4***	173/107/57/14	105/52/19/0	68/55/38/14		173/107/0/0	93/47/0/0	80/60/0/0		0/0/57/14	0/0/33/3	0/0/24/11	
**uCFH/uCr *(ng/mg)***	34.5 (0.0–9220.2)	13 (0.0–34.5)	97.2 (34.7–9220.2)		28.7 (0.0–2305.8)	11.3 (0.0–28.4)	73.9 (28.9–2305.8)		82.1 (0.0–9220.2)	29.5 (0.0–82.7)	611.9 (8.7–9220.2)	
**Histologic*grade*** ^**a**^												
***I/II/III***	34/3/147	26/3/85	8/0/62	<0.001	32/3/135	22/2/73	10/1/62	<0.001	2/0/12	2/0/4	0/0/8	0.77
***IV/V***	131/35	57/5	74/30		99/11	41/2	58/9		32/24	18/12	14/12	
***I+II+III/IV+V***	184/166	114/62	70/104	<0.001	170/110	97/43	73/67	0.003	14/56	6/30	8/26	
**Therapy (%)**												
***ACEIand/ or ARB***	337(96%)	167(94.9%)	170(97.1%)	0.414	269(96%)	133(95.0%)	136(97.1%)	0.356	68(95.8%)	34(94.4%)	34(97.1%)	0.578
***Immuno-suppressive***	158(45%)	58 (33.0%)	100(57.1%)	<0.001	111(96%)	38(27.1%)	73(52.1%)	<0.001	47(66.2%)	23(63.9%)	24(68.6%)	0.558
**Composite events (%)**	40	4(2.3%)	36(20.6%)	<0.001	17	1(0.7%)	16(11.4%)	<0.001	23	11(30.6%)	12(34.3%)	0.741
**Follow-up *(months)***	51.8±26.6	51.7±25.9	51.9±27.4	0.916	53.2±25.8	49.8±25.1	56.5±26.1	0.03	46.6±29.2	52.9±27.4	40.1±30.0	0.064

Data are shown as mean ± SD or median (inter-quartile range) for continuous variables and proportions for categorical variables. Abbreviation: uCFH, urinary complement factor H; uCr, urinary creatinine; MAP, Mean arterial blood pressure; eGFR, estimate glomerular filtration rate; ACE-I, angiotensin-converting enzyme inhibitors; ARB, angiotensin II receptor blocker.

^a^Histological grades were divided into groups A (Hass I, II, and III) and B (Hass IV and V) [25], Pathological data of one patient was lost.

Each patient was followed up at least for 12 months. The renal composite endpoint was defined as: 1) end-stage renal disease (ESRD) (eGFR<15mL/min/ 1.73 m^2^); 2) eGFR decline ≥50% from the baseline value; 3) doubling of plasma creatinine levels.

### Measurement of urinary CFH by Enzyme-linked Immunosorbent Assay (ELISA)

Urine spots were collected on the day of renal biopsy and stored at -80°C until measurement. Urinary CFH concentrations were measured by ELISA described previously, with minor modifications [15]. Briefly, plates (Nalge-Nunc, Rochester, NY) were coated with goat anti-human factor H (Calbiochem, Darmstadt, Germany) at a concentration of 10μg/mL in 0.05 M bicarbonate buffer (pH = 9.6). Plates coated with bicarbonate buffer alone were also prepared simultaneously as antigen-free controls. The reaction volume was 100 μL. Urine samples from patients with IgAN and healthy controls were added to wells in duplicate. After incubation and washing, 1:1000 diluted mouse anti-human CFH (US Biological, Swampscott, MA, USA) was added. Alkaline phosphatase-conjugated goat anti-mouse IgG (Sigma, St. Louis, MO, USA) was added as a second antibody following three washes. The absorbance was measured at 405 nm with an EL312 Bio-Kinetics microplate reader (Bio-Tek Instruments, Inc., Winooski, VT, USA). Commercially available highly purified human factor H was used to establish the standard curve.

The urinary CFH measurements were corrected by urinary creatinine concentration, and the ratios of urinary CFH over urinary creatinine (uCFH/uCr) were used for analysis.

### Detection of CFH deposition by immunofluorescence staining

Renal tissue samples were obtained from IgAN renal biopsy specimens. Paraffin-embedded blocks were cut into sections of 4-μm-thick sections and mounted on pre-coated slides. The paraffin section was deparaffinized with xylene and dehydrated with ethanol. Endogenous peroxidase activity was blocked by incubating sections in a 3% H_2_O_2_ solution at room temperature for 30 min. Antigen retrieval was performed by incubating the sections in pepsin at 37°C for 1h. After blocking with phosphate buffered saline (PBS) containing 3% bovine serum albumin for 30min, the sections were incubated overnight at 4°C with primary goat anti-human CFH antibody (1:8000, diluted in PBS). Bound antibodies were detected with biotinylated rabbit anti-goat IgG. Diaminobenzidine was used as a chromogen.

### Statistical analyses

Baseline features were reported as mean±SD, median (inter-quartile range [IQR]) for continuous variables, and ratios/percentages for categorical data. One-way post-hoc ANOVA using Bonferroni multiple comparison, Chi-square test, Mann-Whitney *U* test, and Kruskal-Wallis test was used to test for differences between groups. Spearman correlation coefficient was used to determine the correlation between urinary CFH levels and clinical variables.

A Cox proportional hazards model was used to analyze the association between urinary CFH levels and renal outcome in the whole group, as well as in subgroups with eGFR≥60 and eGFR<60mL/min/1.73m^2^. Both log (uCFH/uCr) and uCFH/uCrby quartiles and median were analyzed in the Cox models. The lowest group in quartiles or median was defined as the reference group in each analysis. Hazard ratio was tested in unadjusted and three adjusted Cox models. Survival curves were derived from the Kaplan—Meier analysis and compared by log-rank test. Receiver operating characteristic (ROC) analysis was used to assess the predictive power of log(uCFH/uCr).

Proportional hazards assumptions were verified by testing the interaction of survival time and log (uCFH/uCr), uCFH/uCr quartiles, and (low vs. high) uCFH/uCr (*P* = 0.269, *P* = 0.161, and *P* = 0.236, respectively). All statistical tests were performed using SPSS version 13.0.

## Results

### Urinary CFH levels at baseline correlated with clinical/histological parameters

**Fig 1:**
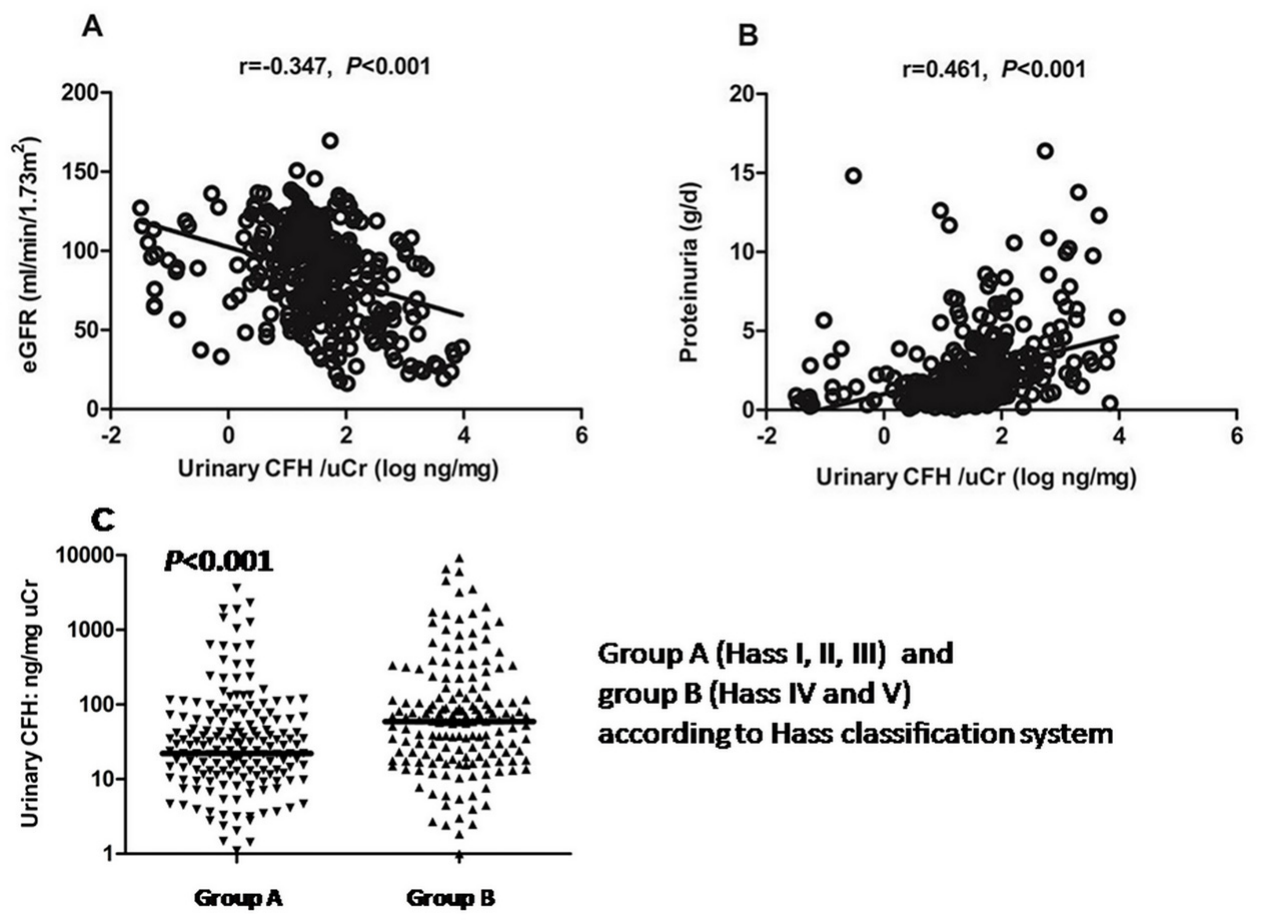
Correlation between urinary CFH levels and clinical/histologic parameters. A. Correlation between urinary CFH levels and baseline eGFR. Urinary CFH levels are shown as log(uCFH/uCr). Spearman correlation analysis was used to determine the correlation between urinary CFH levels and baseline eGFR. Spearman's r = −0.347, *P*<0.001, and after adjusting for baseline proteinuria r = −0.291, *P*<0.001. B. Correlation between urinary CFH levels and baseline proteinuria. Urinary CFH level are shown as log(uCFH/uCr). Spearman r-value and the *P*-value are shown. C. Urinary CFH levels and pathologic changes. Patients were divided into A (Hass I, II and III) and B (Hass IV and V) groups according to the Hass classification system. Urinary CFH levels were compared using the non-parametric Mann-Whitney *U* test.

Urinary CFH levels are shown as uCFH/uCr. The uCFH/uCr (34.5 ng/mg [0.0–9220.2]) at baseline was correlated with baseline eGFR (r = −0.347, *P*<0.001, Fig 1A), baseline proteinuria (r = 0.461, *P*<0.001, Fig 1B) and pathologic changes. After adjusting for baseline proteinuria, urinary CFH levels remained correlated with eGFR (r = −0.291, *P*<0.001). Patients with more diffuse and severe histological lesions (Hass IV and V) had higher urinary CFH levels compared to those with mild focal lesions (Hass I,II, and III) (*P*<0.001) (Fig 1C).

Immunofluorescence staining of CFH showed increased CFH deposition in the glomeruli of patients with IgAN compared to normal controls (S1 Fig).

### Urinary CFH levels associated with renal survival in the whole cohort

Twenty-seven patients (7.7%) developed ESRD, 10 patients (2.8%) had a ≥50%eGFR decline, and 3 patients (0.85%) had doubled plasma creatinine levels during follow-up (51.8±26.6 months).

**Fig 2:**
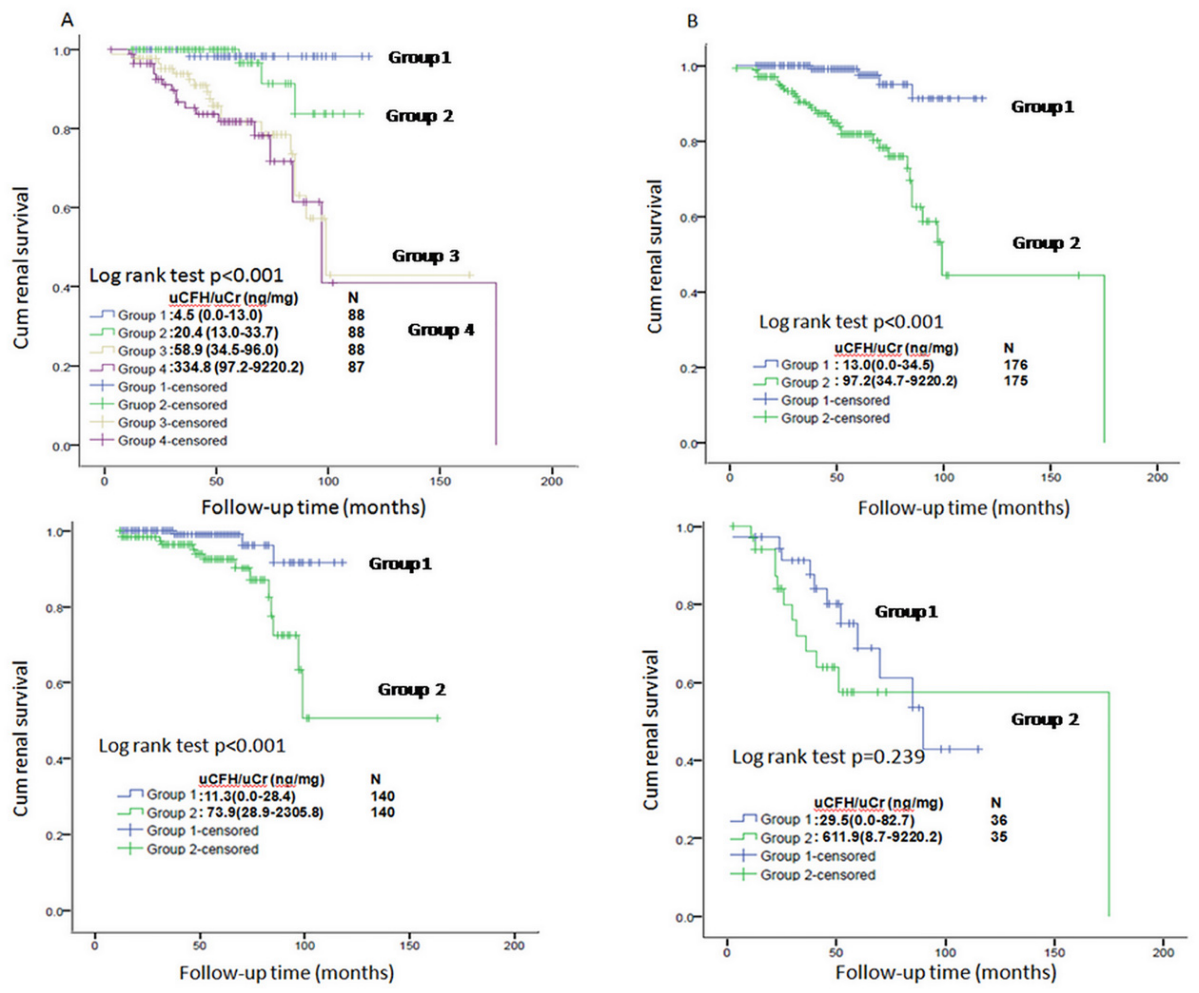
Urinary CFH levels predicted renal survival in the IgAN cohort. The survival curve was derived from Kaplan—Meier analysis and compared by log-rank test. A. Urinary CFH levels and renal survival in the whole cohort by quartiles. Patients were divided into four groups according to uCFH/uCr quartiles; the first quartiles had the lowest uCFH/uCr values (0.0–13.0ng/mg). Patients in the third and fourth quartiles had more frequently progressed to composite endpoints. B. Median urinary CFH levels and renal survival in the whole cohort. All individuals were divided into two groups by median uCFH/uCr values. The patients with higher uCFH/uCr had increased risk of progressing to the composite endpoint. C. Median urinary CFH levels and renal survival in the subgroup of patients with eGFR≥60mL/min/1.73m^2^. Individuals with eGFR≥60mL/min/1.73m^2^ were divided into two groups by median uCFH/uCr. The patients with higher uCFH/uCrhad increased risk of progress to a composite endpoint. D. Median urinary CFH levels and renal survival in the subgroup of patients with eGFR<60mL/min/1.73m^2^.

Patients were divided into four groups by quartiles of uCFH/uCr; patients in the first quartile had the lowest uCFH/uCr values(0.0–13.0ng/mg).Baseline data showed that patients in the third and fourth quartiles had more proteinuria, lower eGFR, diffuse histological lesions, and higher ESRD (Table 2).Univariate analyses revealed that clinical features, including uCFH/uCr, mean arterial pressure (MAP), eGFR, proteinuria, histological grading, and immunosuppressive therapy, were associated with renal outcome(S1 Table).We therefore performed unadjusted and adjusted models in further multivariate Cox’s regression analyses. Model I was adjusted for eGFR, proteinuria and MAP. Histological grading was added to Model II based on Model I, and immunosuppressive therapy was added to the model III based on model II. In all four Cox models, uCFH/uCr was associated with disease progression as continuous and categorical traits. When the lowest quartile uCFH/uCr was selected as a reference group, the risk of kidney function decline increased in the third and fourth quartile (Table 3). Kaplan-Meier curve analysis showed that higher uCFH/uCr ratios predicted worse renal outcome during follow-up (log rank, *P*< 0.001) (Fig 2A).

**Table 2:** Clinical and histological data of four groups defined by uCFH/uCr quartiles.

Group	1	2	3	4	
uCFH/uCr(ng/mg)	4.5(0.0–13.0)	20.4(13.0–33.7)	58.9(34.5–96.0)	334.8(97.2–9220.2)	P value
**Number**	88	88	88	87	
**Gender (M)**	52	43	41	47	0.35
**Age(year)**	31.2±9.8	32.9±10.9	35.0±12.2	35.4±13.5	0.065
**MAP (mmHg)**	93.0±10.0	92.2±11.5	93.4±11.9	95.9±12.1	0.166
**Proteinuria (g/d)**	0.9(0.1–14.8)	1.3(0.03–7.1)	1.7(0.2–8.6)	3.0(0.2–16.4)	<0.001
**eGFR(ml/min/1.73m** ^**2**^ **)**	95.7±24.9	94.1±27.6	83.2±29.5	70.6±29.9	<0.001
**ACEI and/or ARB**	85(96.6%)	82(93.2%)	84(95.5%)	86(98.9%)	0.283
**Immunosuppressive therapy**	27 (30.7%)	31(35.2%)	43(48.9%)	57(65.5%)	<0.001
**Histological grading** ^**a**^					
**(I+II+III/IV+V)**	63/25	51/37	38/50	32/54	<0.001
**Composite events (%)**	1(1.1%)	3(3.4%)	13(14.8%)	23(26.4%)	<0.001

Abbreviation: uCFH, urinary complement factor H; uCr, urinary creatinine; MAP, Mean arterial blood pressure; ACE-I, angiotensin-converting enzyme inhibitors; ARB, angiotensin II receptor blocker; eGFR, estimate glomerular filtration rate; IQR, interquartile range.

^a^Histological grades were divided into groups A (Hass I, II, and III) and B (Hass IV and V) [25].

Continuous data was compared by one-way analysis of variance (ANOVA) or Kruskal-Wallis test; dichotomous and categorical data were analyzed by Chi-square test.

uCFH/uCr value was shown as median (IQR) ng/mg.

**Table 3:** Urinary CFH levels were associated with renal outcome in the whole IgAN cohort.

		Hazard Ratio (95% CI)		
	Unadjusted	Model 1^*a*^	Model 2 ^*b*^	Model 3^*c*^
**Log(uCFH/uCr)**	2.68 (1.81–3.96)	1.84 (1.22–2.78)	1.98 (1.28–3.08)	1.74 (1.16–2.64)
	(*P*<0.001)	(*P* = 0.004)	(*P* = 0.002)	(*P* = 0.008)
**uCFH/uCrquartiles**				
**1**	1 [Reference]	1 [Reference]	1 [Reference]	1 [Reference]
**2**	3.18 (0.33–30.62)	2.49(0.26–24.00)	2.49(0.26–23.95)	2.48 (0.26–23.95)
	(P = 0.316)	(P = 0.429)	(P = 0.431)	(P = 0.431)
**3**	17.23(2.30–129.14)	11.56(1.53–84.47)	11.49(1.52–86.93)	11.48 (1.52–86.93)
	(P = 0.006)	(P = 0.018)	(P = 0.018)	(P = 0.018)
**4**	20.87(2.77–157.18)	12.56(1.64–96.25)	10.71(1.29–88.63)	12.78(1.67–97.82)
	(P = 0.003)	(P = 0.015)	(P = 0.014)	(P = 0.032)
**CFH/uCr**				
**(Low vs. high)**	9.13 (3.24–25.70)	6.59(2.30–18.86)	6.62(2.31–18.95)	6.25 (2.18–17.90)
	(P<0.001)	(P<0.001)	(P<0.001)	(P = 0.001)

Abbreviation: CI, confidence interval; uCFH, urinary complement factor H; uCr, urinary creatinine.

Cox proportional hazard model was used to do the analyses. A composite endpoint was defined asESRD, ≥50%eGFR decline or doubling of serum creatinine levels. Urinary CFH levels wereshownas uCFH/uCr. uCFH/uCr values were used as continuous, dichotomous and quartile variables in the analyses.Adjusted models were set up as below.

^a^Model 1 adjusted for eGFR, proteinuria, mean arterial blood pressure(MAP).

^b^Model 2 adjusted for covariates in model 1 plus histological grades(mild and severe lesion groups).

^c^Model 3 adjusted for covariates in model 2 plus prednisone and/or other immunosuppressive agents.

We also analyzed uCFH/uCr by median in multivariate Cox regression. Briefly, we set up the same models described above and adjusted for the same parameters as in the whole cohort. Higher levels of urinary CFH were also associated with poor renal outcome as continuous and categorical traits (Table 3, Fig 2B)

**Fig 3:**
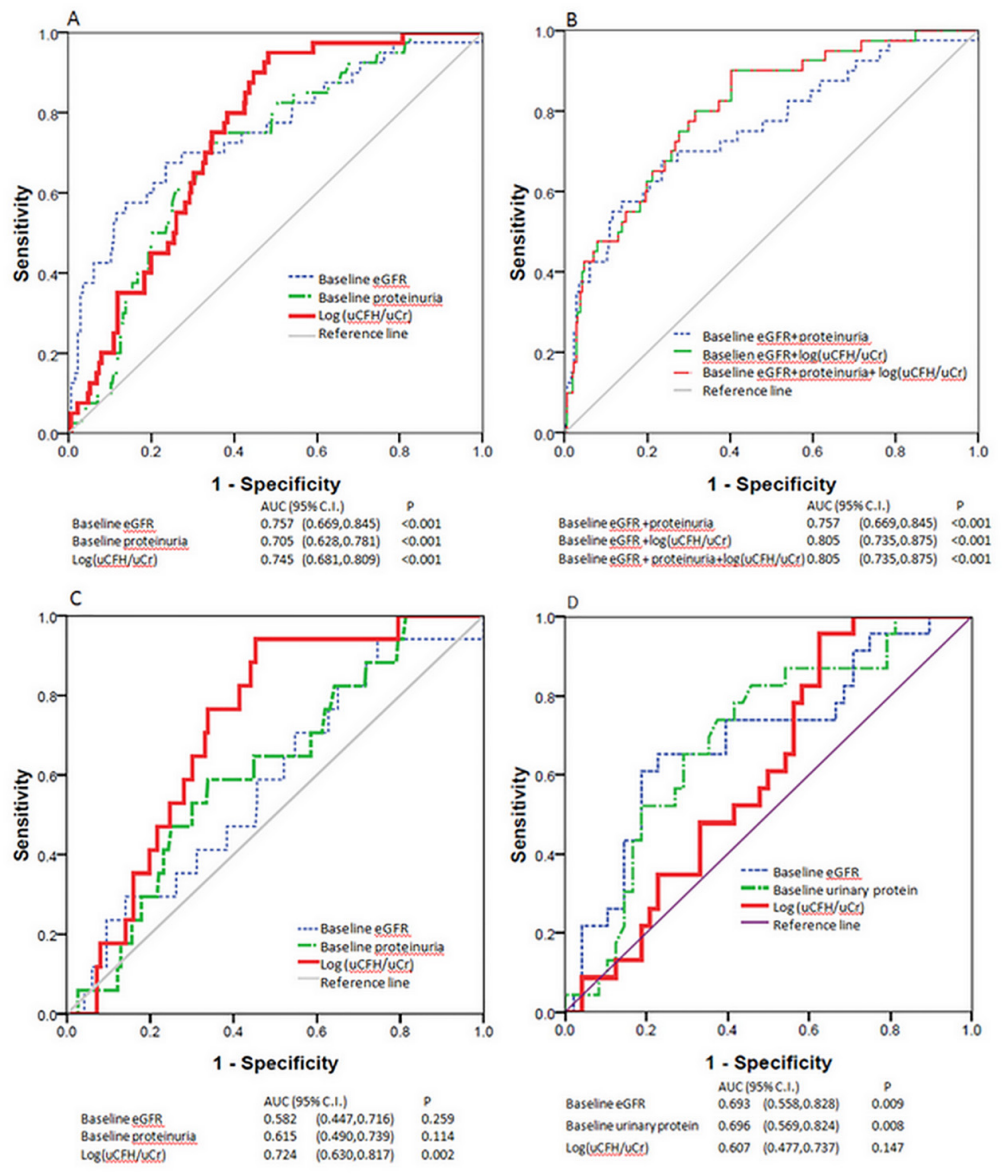
ROC curve analysis of the predictive value for adverse outcomes of eGFR, proteinuria, log(uCFH/uCr) and their combination. ROC: receiver operating characteristic curve; AUC: area under curve. A. ROC for renal outcome in the whole cohort. B. Incorporated ROC for renal outcome in the whole cohort. C. ROC for renal outcome in a subgroup with eGFR≥60mL/min/1.73m^2^. D. ROC for renal outcome in a subgroup with eGFR<60mL/min/1.73m^2^.

Then we separately calculated the area under curve (AUC) for log(uCFH/uCr), eGFR and proteinuria. The AUC for eGFR and proteinuria were 0.757and 0.705, respectively. Log(uCFH/uCr) was not more predictive than eGFR or proteinuria (AUC = 0.745). However the AUC increased to 0.805 after we incorporated log(uCFH/uCr), eGFR and proteinuria (Fig 3A and 3B).

### Predictive value of urinary CFH levels in the subgroup with eGFR≥60 mL/min/1.73m^2^

We also explored whether urinary CFH levels predicted renal outcome in subgroups in early stages of CKD (eGFR≥60 mL/min/1.73m^2^).

Baseline data for the subgroup are listed in Table 1. Fewer patients reached the composite end point compared with the whole group. Only 17patients reached the composite endpoint (ESRD, eGFR decline ≥50% of baseline, or doubling of plasma creatinine levels).

Because of limited number of patients that reached the composite endpoint, we only analyzed median uCFH/uCr in multivariate Cox regression. Briefly, we set up the same models and adjusted the same parameters as for the whole cohort. Higher levels of urinary CFH were also associated with poor renal outcome as continuous and categorical traits (Table 4).

**Table 4:** Urinary CFH levels were associated with renal outcome in the subgroups.

	Unadjusted	Model 1^*a*^	Model 2 ^*b*^	Model 3^*c*^
		Hazard Ratio (95% CI)		
***eGFR≥60 ml/min/1*.*73m*** ^***2***^				
**Log(uCFH/uCr)**	2.21 (1.13–4.30)	2.21 (1.13–4.30)	2.21 (1.13–4.3)	2.21(1.13–4.30)
	(*P* = 0.019)	(*P* = 0.019)	(*P* = 0.019)	(*P* = 0.019)
**uCFH/uCr**	12.75(1.69–96.24)	12.75(1.69–96.24)	12.75(1.69–96.24)	12.75(1.69–96.24)
**(Low vs. high)**	(*P* = 0.014)	(*P* = 0.014)	(*P* = 0.014)	(*P* = 0.014)
***eGFR<60 ml/min/1*.*73m*** ^***2***^				
**Log(uCFH/uCr)**	1.81(1.11–2.96)			
	(*P* = 0.017)	(*P* = 0.509)	(*P* = 0.385)	(*P* = 0.385)
**uCFH/uCr**				
**(Low vs. high)**	(*P* = 0.203)	(*P* = 0.801)	(*P* = 0.721)	(*P* = 0.721)

Abbreviation: CI, confidence interval; uCFH, urinary complement factor H; uCr, urinary creatinine.

Cox proportional hazard model was used to do the analyses. A composite endpoint was defined asESRD, ≥50%eGFR decline or doubling of serum creatinine levels. Urinary CFH levels were shown as uCFH/uCr. uCFH/uCr values were used as continuous, dichotomous and quartile variables in the analyses. Adjusted models were set up as below.

^a^Model 1 adjusted for eGFR, proteinuria, mean arterial blood pressure(MAP).

^b^Model 2 adjusted for covariates in model 1 plus histological grades (mild and severe lesion groups).

^c^Model 3 adjusted for covariates in model 2 plus prednisone and/or other immunosuppressive agents.

Kaplan-Meier analysis also showed that patients with higher uCFH/uCr values had obvious lower renal survival (log rank, *P*< 0.001) (Fig 2C).

ROC curve analysis (Fig 3C), showed that log(uCFH/uCr) had a better predictive value (AUC = 0.724, *P* = 0.002) for renal outcome at early stage (eGFR≥60 mL/min/1.73m^2^) compared to baseline eGFR (AUC = 0.582, *P* = 0.259) and proteinuria (AUC = 0.615, *P* = 0.114).

We also investigated whether urinary CFH levels predicted renal outcome in a subgroup of patients with eGFR<60 mL/min/1.73m^2^.Urinary CFH levels were not associated with renal outcome in the 70 patients in this subgroup (Table 4 and Figs 2D and 3D).

## Discussion

In this study, we measured urinary CFH levels in an IgAN cohort and found that higher urinary CFH levels were associated with lower eGFR, heavier proteinuria and diffuse pathological changes, similar to our previous study findings [15]. We also observed in the current study that baseline urinary CFH levels predicted IgAN progression as either continuous or categorical traits, in the whole cohort as well as the subgroup with eGFR≥60mL/min/1.73m^2^. ROC curve analysis demonstrated that high levels of urinary CFH had a similar predictive value for renal outcome with baseline eGFR and proteinuria in the whole cohort. Subgroup analysis of patients with normal eGFR ≥60 mL/min/1.73m^2^, revealed that urinary CFH showed a better predictive value compared with eGFR or proteinuria. In the subgroup with eGFR<60 mL/min/1.73m^2^, urinary CFH level were not associated with renal outcome, and renal survival was determined by eGFR.

The complement system plays an important role in IgAN. Deposition of IgA1 in mesangial areas is usually accompanied by complements including C3, C4d, C4-binding protein, mannose-binding lectin, C5b-9, and properdin [5,7,19,20,21]. The deposited immune complexes can activate alternative and lectin pathways [2,3,4,5,6,7,8,9,10], and initiate inflammatory process. As a crucial factor that accelerates C3bdegradation, CFH deposition in mesangial areas increases with complement activation [1]. Urinary CFH levels also increased with lower eGFR, larger proteinuria and severe pathological lesions, thus reflecting disease activity [13,14,15].

We identified that baseline urinary CFH levels, a correlate of the inflammatory process, also predicted IgAN progression. A recent study also found that decreased circulating C3 levels and mesangial C3 deposition was associated with renal outcome in patients with IgAN [4]. Both studies supported the crucial roles of complements during the disease process, and measurement of complement factors in fluid or renal tissue could be a good marker for disease activity, prognosis, and even inform treatment with immunosuppressive agents. However detection of such markers in urine is easier, less influenced by other factors and can be repeated during extended patient follow up. IgAN repeats IgA deposition, complement activation, inflammatory status and fibrosis during its progression to ESRD. CFH, in its role as a complement-related factor, may be a good marker and alternative to repeated renal biopsy to guide treatment. However only baseline urinary CFH levels were measured in this study. Future studies should measure the variation of urinary CFH levels during patient follow-up and evaluate the effectiveness of immunosuppressive treatments to decrease urinary CFH levels and thus, improve renal outcome.

In previous studies, impaired renal function was identified as the most powerful predictive factor of renal outcome [22,23,24,25,26]. However, patients with IgAN and impaired renal function are usually in a later disease stage, and are less likely to receive appropriate and timely treatment. Thus, it is necessary to identify biomarkers that can distinguish IgAN patients with poor prognosis at early stages of IgAN, when eGFR is normal. In this study, we also observed that higher urinary CFH levels increased the risk of renal function decline in the subgroup of patients with normal eGFR. ROC analysis showed that log(uCFH/uCr) levels in IgAN had better predictive value compared with eGFR or proteinuria when eGFR≥60 mL/min/1.73m^2^. Therefore in addition to proteinuria, higher urinary levels of CFH may be of concerned in early stage of IgAN, and urinary CFH levels may be better indicators of inflammation status than the presence of proteinuria.

Although 351 patients were involved in this study, they were all from a single center. Since IgAN is a renal disease with slow progression, and individuals undergoing renal biopsy were usually in the early stages, few patients reached the composite endpoint in this study, especially in the subgroup with eGFR> = 60mL/min/1.73m^2^, which might result in false-positive or false-negative results. A longer follow-up time and lager cohort from additional centers are needed to validate these findings.

In conclusion, we found urinary CFH levels to be independently associated with progression of IgAN, and urinary CFH may be a valuable factor to predict progress of IgAN. These findings suggest that complement activation may be involved in the IgAN pathogenesis.

## Supporting Information

S1 Fig Immunofluorescence staining of CFH in patients with IgAN. (TIF)

S1 Table Unadjusted risk estimates by Cox’s proportional hazard models for the composite endpoint in IgAN patients. (DOCX)

## References

[1] Rodriguez de Cordoba S, Esparza-Gordillo J, Goicoechea de Jorge E, Lopez-Trascasa M, Sanchez-Corral P. The human complement factor H: functional roles, genetic variations and disease associations. MolImmunol. 2004;41: 355–367. 15163532 10.1016/j.molimm.2004.02.005

[2] Endo M, Ohi H, Ohsawa I, Fujita T, Matsushita M. Glomerular deposition of mannose-binding lectin (MBL) indicates a novel mechanism of complement activation in IgA nephropathy. Nephrol Dial Transplant. 1998;13: 1984–1990. 9719152 10.1093/ndt/13.8.1984

[3] Hiemstra PS, Gorter A, Stuurman ME, Van Es LA, Daha MR. Activation of the alternative pathway of complement by human serum IgA. Eur J Immunol.1987;17: 321–326. 3569402 10.1002/eji.1830170304

[4] Kim SJ, Koo HM, Lim BJ, Oh HJ, Yoo DE, Shin DH, et al Decreased circulating C3 levels and mesangial C3 deposition predict renal outcome in patients with IgA nephropathy. PLoS One. 2012;7: e40495 10.1371/journal.pone.0040495 22792353 PMC3391269

[5] Nakagawa H, Suzuki S, Haneda M, Gejyo F, Kikkawa R. Significance of glomerular deposition of C3c and C3d in IgA nephropathy. Am J Nephrol.2000;20: 122–128. 10773612 10.1159/000013568

[6] Roos A, Bouwman LH, van Gijlswijk-Janssen DJ, Faber-Krol MC, Stahl GL, Daha MR. Human IgA activates the complement system via the mannan-binding lectin pathway. J Immunol.2001;167: 2861–2868. 11509633 10.4049/jimmunol.167.5.2861

[7] Roos A, Rastaldi MP, Calvaresi N, Oortwijn BD, Schlagwein N, van Gijlswijk-Janssen DJ, et al Glomerular activation of the lectin pathway of complement in IgA nephropathy is associated with more severe renal disease. J Am SocNephrol. 2006;17: 1724–1734. 16687629 10.1681/ASN.2005090923

[8] Wyatt RJ, Kanayama Y, Julian BA, Negoro N, Sugimoto S, Hudson EC, et al Complement activation in IgA nephropathy. Kidney Int. 1987;31: 1019–1023. 3586493 10.1038/ki.1987.101

[9] Zhang W, Lachmann PJ. Glycosylation of IgA is required for optimal activation of the alternative complement pathway by immune complexes. Immunology.1994;81: 137–141. 8132210 PMC1422296

[10] Zwirner J, Burg M, Schulze M, Brunkhorst R, Gotze O, Koch KM, et al Activated complement C3: a potentially novel predictor of progressive IgA nephropathy. Kidney Int. 1997;51: 1257–1264. 9083294 10.1038/ki.1997.171

[11] Gharavi AG, Kiryluk K, Choi M, Li Y, Hou P, Xie J, et al Genome-wide association study identifies susceptibility loci for IgA nephropathy. Nat Genet. 2011;43: 321–327. 10.1038/ng.787 21399633 PMC3412515

[12] Onda K, Ohi H, Tamano M, Ohsawa I, Wakabayashi M, Horikoshi S, et al Hypercomplementemia in adult patients with IgA nephropathy. J Clin Lab Anal.2007;21: 77–84. 17385664 10.1002/jcla.20154 PMC6649110

[13] Onda K, Ohsawa I, Ohi H, Tamano M, Mano S, Wakabayashi M, et al Excretion of complement proteins and its activation marker C5b-9 in IgA nephropathy in relation to renal function. BMC Nephrol. 2011; 12: 64 10.1186/1471-2369-12-64 22111871 PMC3283454

[14] Tamano M, Fuke Y, Endo M, Ohsawa I, Fujita T, Ohi H. Urinary complement factor H in renal disease. Nephron. 2002; 92: 705–707. 12372960 10.1159/000064090

[15] Zhang JJ, Jiang L, Liu G, Wang SX, Zou WZ, Zhang H, et al Levels of urinary complement factor H in patients with IgA nephropathy are closely associated with disease activity. Scand J Immunol. 2009 69: 457–464. 10.1111/j.1365-3083.2009.02234.x 19508377

[16] Levey AS, Stevens LA, Schmid CH, Zhang YL, Castro AF 3rd, Feldman HI, et al A new equation to estimate glomerular filtration rate. Ann Intern Med. 2009;150: 604–612. 19414839 10.7326/0003-4819-150-9-200905050-00006 PMC2763564

[17] Haas M. Histologic subclassification of IgA nephropathy: a clinicopathologic study of 244 cases. Am J Kidney Dis. 1997;29: 829–842. 9186068 10.1016/s0272-6386(97)90456-x

[18] Zhao N, Hou P, Lv J, Moldoveanu Z, Li Y, Kiryluk K, et al The level of galactose-deficient IgA1 in the sera of patients with IgA nephropathy is associated with disease progression. Kidney Int. 2012;82: 790–796. 10.1038/ki.2012.197 22673888 PMC3443545

[19] Rauterberg EW, Lieberknecht HM, Wingen AM, Ritz E. Complement membrane attack (MAC) in idiopathic IgA-glomerulonephritis. Kidney Int. 1987;31: 820–829. 3573542 10.1038/ki.1987.72

[20] Stangou M, Alexopoulos E, Pantzaki A, Leonstini M, Memmos D. C5b-9 glomerular deposition and tubular alpha3beta1-integrin expression are implicated in the development of chronic lesions and predict renal function outcome in immunoglobulin A nephropathy. Scand J UrolNephrol. 2008;42: 373–380. 10.1080/00365590801943241 19230171

[21] Tomino Y, Sakai H, Nomoto Y, Endoh M, Arimori S, Fujita T. Deposition of C4-binding protein and beta 1H globulin in kidneys of patients with IgA nephropathy. Tokai J ExpClin Med. 1981;6: 217–222. 6458121

[22] Bogenschutz O, Bohle A, Batz C, Wehrmann M, Pressler H, Kendziorra H, et al IgA nephritis: on the importance of morphological and clinical parameters in the long-term prognosis of 239 patients. Am J Nephrol.1990;10: 137–147. 2349957 10.1159/000168068

[23] D'Amico G.Natural history of idiopathic IgA nephropathy: role of clinical and histological prognostic factors. Am J Kidney Dis. 2000;36: 227–237. 10922300 10.1053/ajkd.2000.8966

[24] Frimat L, Briancon S, Hestin D, Aymard B, Renoult E, Huu TC, et al IgA nephropathy: prognostic classification of end-stage renal failure. L'Association des Nephrologues de l'Est.Nephrol Dial Transplant. 1997;12: 2569–2575. 9430853 10.1093/ndt/12.12.2569

[25] Koyama A, Igarashi M, Kobayashi M. Natural history and risk factors for immunoglobulin A nephropathy in Japan. Research Group on Progressive Renal Diseases. Am J Kidney Dis. 1997;29: 526–532. 9100040 10.1016/s0272-6386(97)90333-4

[26] Radford MG Jr, Donadio JV Jr, Bergstralh EJ, Grande JP. Predicting renal outcome in IgA nephropathy. J Am SocNephrol. 1997;8: 199–207. 9048338 10.1681/ASN.V82199

