# Supplementary figures and images for: Implication of Urinary Complement Factor H in the Progression of Immunoglobulin A Nephropathy
Source: PLoS One. 2015 Jun 2;10(6):e0126812. doi: 10.1371/journal.pone.0126812 (PMC4452759; doi:10.1371/journal.pone.0126812)

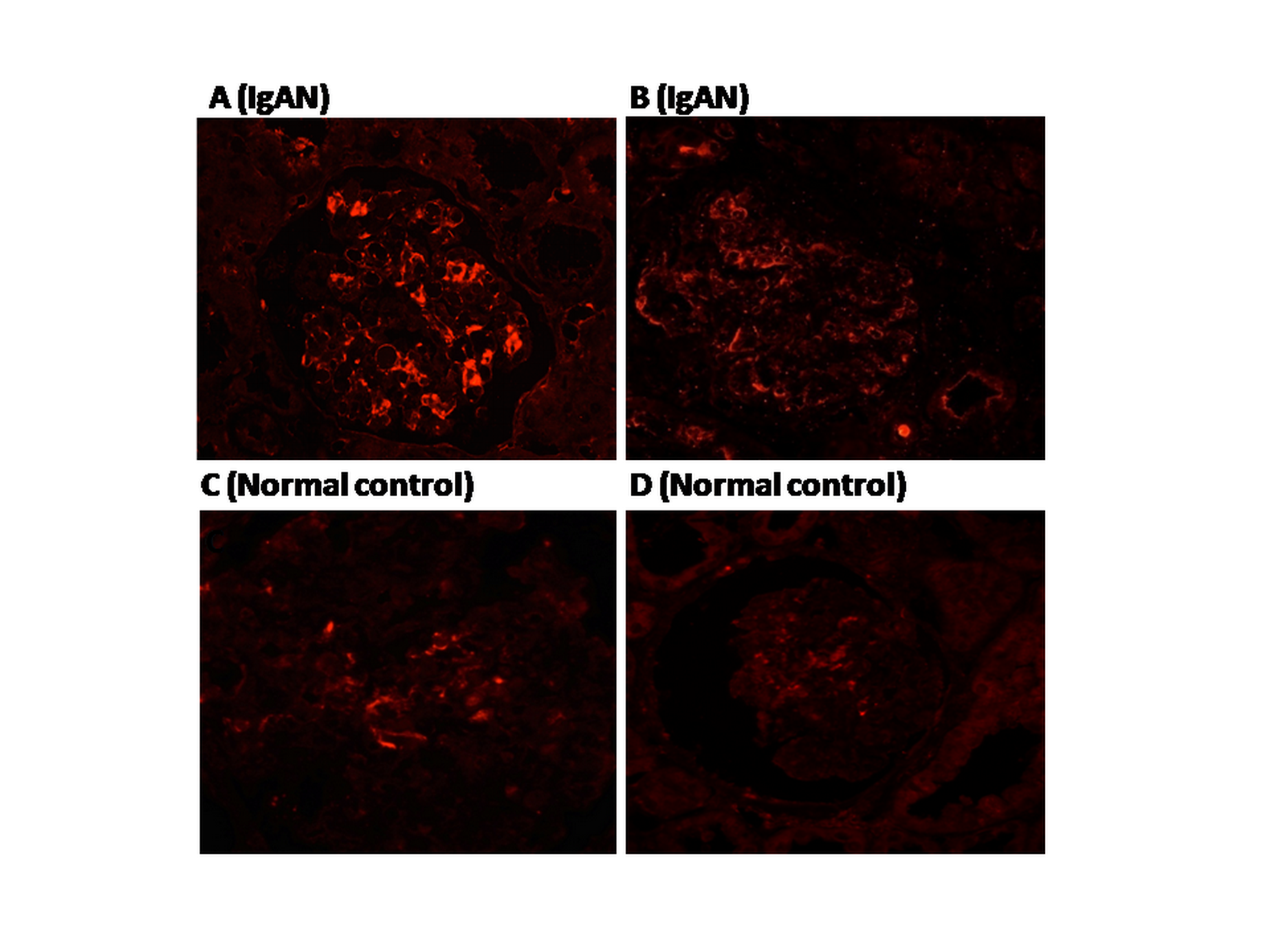

Supplement: S1 Fig — (TIF) [file pone.0126812.s001.tif]
