# Supplementary material for: Implication of Urinary Complement Factor H in the Progression of Immunoglobulin A Nephropathy
Source: PLoS One. 2015 Jun 2;10(6):e0126812. doi: 10.1371/journal.pone.0126812 (PMC4452759; doi:10.1371/journal.pone.0126812)
Supplement: S1 Table — (DOCX) [file pone.0126812.s002.docx]

**Supporting Information Table S1. Unadjusted risk estimates by Cox’s proportional hazard models for the composite endpoint in IgAN patients.**

| **Risk factor** | **Univariate** | |
| --- | --- | --- |
|  | **Hazard Ratio (95% CI)** | ***P*-value** |
| **Gender** | 1.08 (0.57–2.02) | 0.820 |
| **Age(year)** | 0.99(0.96–1.02) | 0.412 |
| **MAP(mm Hg)** | 1.03(1.00–1.05) | 0.041 |
| **Proteinuria (g/d)** | 1.18(1.08–1.29) | <0.001 |
| **eGFR (ml/min/1.73m2)** | 0.96(0.95–0.98) | <0.001 |
| **Log(uCFH/uCr)** | 2.68 (1.81–3.96) | <0.001 |
| **uCFH/uCr quartiles** |  |  |
| **1** | 1 [Reference] |  |
| **2** | 3.16 (0.33–30.62) | 0.316 |
| **3** | 17.23 (2.30–129.14) | 0.006 |
| **4** | 20.87 (2.77–157.18) | 0.003 |
| **uCFH/uCr** |  |  |
| **(Low vs. high)** | 9.13 (3.24–25.70) | <0.001 |
| **Histological grading( I+II+III/ IV+V )** | 4.41(1.94–10.02) | <0.001 |
| **ACEI and/or ARB** | 0.26 (0.08–0.84) | 0.025 |
| **Prednisone and/or other immunosuppressive agents** | 3.33(1.65–6.71) | 0.001 |

Abbreviation: MAP, Mean arterial blood pressure; ACE-I, angiotensin-converting enzyme inhibitors; ARB, angiotensin II receptor blocker; eGFR, estimate glomerular filtration rate. Urinary CFH level was showed as uCFH/uCr. uCFH/uCr was analyzed as continuous trait, dichromatic and quartile categorical variables. Histological grading was divided into group (Haas I to III) and severe lesions group (Haas IV and V) .
